# Influence of *in vitro *supplementation with lipids from conventional and Alpine milk on fatty acid distribution and cell growth of HT-29 cells

**DOI:** 10.1186/1476-511X-10-131

**Published:** 2011-08-04

**Authors:** Christian Degen, Alfred Lochner, Sylvia Keller, Katrin Kuhnt, Sven Dänicke, Gerhard Jahreis

**Affiliations:** 1 Friedrich-Schiller-University, Institute of Nutrition, Dept. of Nutritional Physiology, Dornburger Str. 24, Jena, Germany; 2 Institute of Animal Nutrition, Friedrich Loeffler Institute (FLI), Federal Research Institute for Animal Health, Braunschweig, Germany

**Keywords:** Conjugated linoleic acid, milk lipids, cellular lipid distribution, cancer cells, HT-29

## Abstract

**Background:**

To date, the influence of milk and dairy products on carcinogenesis remains controversial. However, lipids of ruminant origin such as conjugated linoleic acids (CLA) are known to exhibit beneficial effects *in vitro *and *in vivo*. The aim of the present study was to determine the influence of milk lipids of different origin and varying quality presenting as free fatty acid (FFA) solutions on cellular fatty acid distribution, cellular viability, and growth of human colon adenocarcinoma cells (HT-29).

**Methods:**

FAME of conventional and Alpine milk lipids (ML_con_, ML_alp_) and cells treated with FFA derivatives of milk lipids were analyzed by means of GC-FID and Ag^+^-HPLC. Cellular viability and growth of the cells were determined by means of CellTiter-Blue^®^-assay and DAPI-assay (4',6-diamidino-2-phenylindole dihydrochloride), respectively.

**Results:**

Supplementation with milk lipids significantly decreased viability and growth of HT-29 cells in a dose- and time-dependent manner. ML_alp _showed a lower SFA/MUFA ratio, a 8 fold increased CLA content, and different CLA profile compared to ML_con _but did not demonstrate additional growth-inhibitory effects. In addition, total concentration and fatty acid distribution of cellular lipids were altered. In particular, treatment of the cells yielded highest amounts of two types of milk specific major fatty acids (μg FA/mg cellular protein) after 8 h of incubation compared to 24 h; 200 μM of ML_con _(C16:0, 206 ± 43), 200 μM of ML_alp _(C18:1 *c*9, (223 ± 19). Vaccenic acid (C18:1 *t*11) contained in milk lipids was converted to *c*9,*t*11-CLA in HT-29 cells. Notably, the ratio of *t*11,*c*13-CLA/*t*7,*c*9-CLA, a criterion for pasture feeding of the cows, was significantly changed after incubation for 8 h with lipids from ML_alp _(3.6 - 4.8), compared to lipids from ML_con _(0.3 - 0.6).

**Conclusions:**

Natural lipids from conventional and Alpine milk showed similar growth inhibitory effects. However, different changes in cellular lipid composition suggested a milk lipid-depending influence on cell sensitivity. It is expected that similar changes may also be evident in other cell lines. To our knowledge, this is the first study showing a varied impact of complex milk lipids on fatty acid distribution in a colon cancer cell line.

## Background

The incidence for colon cancer world-wide is increasing; the Western-style diet in particular is considered to be one of the main risk factors involved in colon cancer development [1]. In general, the Western-style diet associated with a high fat intake is considered to be an adverse factor. However, the composition of dietary fat is decisive for an assessment of its nutritional value and its role in the incidence of cancer [2]. The nutritional role of milk, dairy products and meat of ruminant origin also displays this controversy since on the one hand, these products contain potent chemopreventive and anticarcinogenic compounds such as branched-chain fatty acids (BCFA), phospholipids, ether lipids, vitamin D, and conjugated linoleic acid (CLA) which play a protective role in colorectal cancer [3,4]. On the other hand, milk lipids contain high amounts of saturated fatty acids (SFA) that are associated with an increased risk of nutrition-related diseases [5]. However, recent data supports a role of dietary fat of animal origin with an increased risk of breast and pancreatic cancer [6,7].

Concerning CLA, however, no significant association between dietary intake and risk of breast cancer has been found according to a recent study [8]. CLA from ruminant origin exhibit an increased potential for anticarcinogenic activity *in vitro *and *in vivo *[9]. CLA (C18:2; *c*/*t, t*/*c, c*/*c, t*/*t*) display a high variation of positional and geometrical isomers with conjugated double bonds depending on the ruminant species, season and the type of feeding. *C*9,*t*11-CLA is the main dietary isomer (90%). In cows, extensive feeding on herbs and pasture with a high composition of PUFA (polyunsaturated fatty acid) increased total CLA amount and CLA precursor such as C18:1 *t*11 (VA, vaccenic acid) in milk fat, and generated different isomers such as *t*11,*c*13-CLA [10]. An intensive feeding diet leads to lower CLA content [11]. To date, only a few studies have investigated the impact of CLA in its natural form, e.g. in butter fat or beef tallow *in vivo *[12-14], and in milk fat or beef extracts *in vitro *on cancer [15-17]. Investigations of nutritional substances relevant to health and diseases in *in vitro *models allow a better understanding of potential effects and the related molecular mechanisms. Remarkably, none of the *in vitro *studies examined the incorporation and metabolism of lipid mixtures into cell lipids to determine whether growth inhibition is associated with fatty acid (FA) distribution. Dietary PUFA, including CLA, have been shown to be effective inducers of cell death [18]. Thus, in this study, we examined the effects of Alpine milk fat naturally enriched in CLA and other PUFA in comparison to conventional milk fat on viability, growth and cellular FA distribution of highly transformed HT-29 colon adenocarcinoma cells. This *in vitro *study model allows an examination of the positive or negative influence of a complete FA spectrum on human health. By supplementation with mixtures containing *c*9,*t*11-CLA and other PUFA as anticarcinogenic compounds, this study aims to clarify the effects of these lipids on colon cancer cells. Previous study models employed these lipids as pure single isomers. In contrast, herein, complex milk lipids from two different origins and of varying quality were used to examine their degree and relevant mode of action.

## Methods

### Milk samples and fatty acid analysis

During the interventional trial, conventional milk lipids (ML_con_, Braunschweig, Germany) were derived from German Holstein cows receiving a diet consisting of 50% concentrate and 50% roughage [19]. Alpine milk lipids (ML_alp_, Switzerland) were prepared from the milk of Simmental x Red Holstein cows kept on summer pasture without concentrate in the Swiss Alps. At least 8 milk lipid samples from each breed were pooled to obtain sufficient free fatty acid (FFA) derivative sample material for the *in vitro *investigation in HT-29 cells. FA distribution of the samples was analyzed after transesterfication of total milk lipids to fatty acid methylesters (FAME) using methanolic sodium methylate 0.5 N at room temperature for 20 min. The FA analysis of total FAME including the distribution of *cis*/*trans*-C18:1 isomers required two GC methods. Firstly, FA distribution of four to twenty-six chain carbon atoms including total CLA was analyzed using a fused-silica capillary column with medium polarity (DB-225 MS: 60 m × 0.25 mm i.d. with 0.25 μm film thickness; Agilent Technologies, USA) as described previously [20]. Secondly, a fused-silica capillary column with high polarity (CP-select: 200 m × 0.25 mm i.d. with 0.25 μm film thickness; Varian, Netherlands) was used to separate octadecenoic acid methylesters with *cis*- and *trans*-configuration under isothermal conditions at 176°C. The temperature of the injector and the flame ionization detector was set at 260°C and 270°C for both GC programs, respectively. Hydrogen was used as a carrier gas. Evaluation of the chromatograms was completed according to the retention time of known standards with GC solution software (GC-solution version 2.3 from Shimadzu, Kyoto, Japan). FAME standards for GC analysis were received from CPS Chemie (Aachen, Germany), Nu-Chek Prep (Elysian, USA), Sigma (Steinheim, Germany), and Supelco (Taufkirchen, Germany). Additionally, silver ion-HPLC (Ag^+^-HPLC) was used to separate FAME of CLA isomers as described previously [21]. In brief, LC10A-HPLC (Shimadzu, Kyoto, Japan) with three silver ion-impregnated columns (Chrompack ChromSpher 5 Lipids; each: 250 mm × 4.6 mm i.d., 5 μm; Varian, Netherlands) was used in series followed by the UV spectrophometric detector SPD-10A at 233 nm (Shimadzu, Kyoto, Japan). Isocratic mobile phase containing hexane/diethyl ether/acetonitril/2-propanol (99.35/0.5/0.1/0.05, v:v:v:v) served as eluent at a flow rate of 1 mL/min.

### Preparation of milk samples for *in vitro *experiments

Investigation with natural complex lipids *in vitro *requires the presence of FFA (free fatty acids), since cells cannot absorb triacylglycerides (TAG) [22]. Against this background, preparation of FFA was conducted by means of an adapted procedure originally described by Bligh and Dyer [23]. Milk lipids (100 mg) were dissolved in 0.75 mL ethanol (96%) and TAG were saponified with 0.75 mL of ethanolic sodium hydroxide 2 N at 70°C for 30 min. The solution was neutralized with 1.5 mL of hydrochloric acid 1 N. FFA were extracted using 1.5 mL chloroform for 5 min under shaking. Two phases were achieved after adding 1.35 mL of 2% sodium chloride solution and 1.5 mL chloroform. After further shaking (5 min) and centrifugation (2300 × *g*), the lower phase was collected. Success of this process was confirmed by thin layer chromatography using hexane/diethyl ether/formic acid (80/20/2, v:v:v). Distribution and amount of FFA were verified. After transesterfication to FAME with 2 mL of a methanolic boron trifluoride solution (5%, wt:wt; 100°C, 2 min), the distribution of FFA was analyzed by means of the two above-mentioned GC methods. Additionally, FA including C16:0, C18:0 and C18:1 *c*9 were absolutely quantified by GC-FID (60 m column) using the TAG tritricosanoin as internal standard (CPS Chemie, Aachen, Germany). Thereafter, total FFA levels were adjusted to 100 mM in ethanol. Finally, the FA distribution and amounts of FFA-ML_alp/con _in ethanol were measured by GC-FID as described above. Before usage in cell culture experiments, FFA-ML_alp/con _solutions were diluted with cell culture medium to 10 mM.

### Cell culture

Human colon adenocarcinoma cells HT-29 were obtained from the American Tissue Culture Collection (ATCC) and maintained in Dulbecco's Modified Eagle's Medium (DMEM; 4.5 g/L glucose, 58 mg/L L-glutamine, without sodium pyruvate; Invitrogen, Darmstadt, Germany) supplemented with fetal bovine serum (10%, v:v; Biochrom, Berlin, Germany). Cells were cultivated as subconfluent monolayers in cell flasks (T-25 cm^2 ^and T-75 cm^2^; Greiner Bio one, Frickenhausen, Germany) under standard conditions at 37°C in a humidified culture incubator with 5% carbon dioxide and 95% humidity.

### Cell viability and growth parameters of HT-29 cells

To obtain results of cell viability and growth parameters, 8000 cells/well were seeded onto a 96-well plate (Greiner Bio one, Frickenhausen, Germany) and were allowed to attach for 24 h. Cells were then treated with two different types of complex milk lipids (FFA-ML_alp/con_). Freshly prepared FFA solutions were added to a final volume of 200 μL culture medium. Concentrations of ML_con _and ML_alp _ranging from 5-250 μM were chosen and incubated for 24 h, 48 h and 72 h and cell viability and growth parameters were determined by means of the CTB assay (CellTiter-Blue^®^; Promega, Mannheim, Germany) and the DAPI assay (4',6-diamidino-2-phenylindole dihydrochloride; Carl Roth GmbH, Karlsruhe, Germany), respectively as previously described [24]. Results of cell viability and growth were calculated on the basis of percentage change to medium control containing 0.25% ethanol.

### Fatty acid analysis after treatment with milk lipids

Following incubation with 100 μM and 200 μM of ML_alp/con _for 8 h and 24 h in 25 cm^2 ^flasks, cells were washed with phosphate-buffered saline (PBS), detached from the flask by a trypsin/EDTA solution (0.05%/0.02%) and centrifuged at 700 × *g *for 5 min at 8°C. After discarding the supernatant the cell pellet was resolved in PBS. Protein content was determined by employing the Bradford method using bovine serum albumin as standard [25]. Cellular lipids were extracted with a methanol/chloroform mixture according to Bligh and Dyer [23]. Tritricosanoin was used as an internal standard. Derivatization of total lipids was conducted with 1 mL methanolic sodium hydroxide 0.5 N (100°C, 10 min) followed by addition of 1 mL of a methanolic boron trifluoride solution (10%, wt:wt; 100°C, 2 min). FAME were extracted three times with hexane (1 × 2 mL, 2 × 1 mL). Relative and absolute quantitative analyses of FAME extracts including CLA distribution by means of GC-FID and Ag^+^-HPLC were performed as described in the section milk samples and FA analysis.

### Statistical analysis

All statistical analyses were performed using SPSS statistics, version 17.0 (^©^2009 SPSS Inc, Illinois, USA). Results were presented as means ± SD. Significant effects of ML_alp/con _on cell viability and cell growth were assessed by one-way ANOVA followed by Dunnett's *post hoc *test (comparison to control group). The multiple comparisons were conducted with two-way ANOVA (milk type × FA-concentration) followed by Tukey's *post hoc *test to compare the results of FA analyses after different treatments. A value of *P *< 0.05 was taken to indicate significance.

## Results

### Characterisation of milk lipids

The FA distribution of ML_con _and ML_alp _showed significant differences depending on the types of milk lipids required to achieve ethanolic FFA-ML_con/alp _solutions (Table 1). FA distribution of ML_con _showed high amounts of SFA (77.7%) and MUFA (monounsaturated fatty acid) (19.9%) and was characterized by low levels of PUFA (2.4%) including 0.3% of total CLA. In contrast, FA distribution of ML_alp _was characterized by decreased quantities of SFA (63.0%), higher concentrations of MUFA (30.3%), and PUFA (6.8%) including 3.2% of total CLA and, therefore, showed a significantly higher ratio of PUFA/SFA compared to ML_con_. However, concentrations of n-6 PUFA were similar to ML_con_, whereas n-3 PUFA, C18:1 *t*11 and CLA were significantly higher by factors of 3, 10 and 9, respectively (Table 1A). Generally, the nutritional properties of the milk lipids were similar with regards to elevated levels of SFA. In contrast, since ML_alp _comprised a significantly higher unsaturated character, we considered this milk as being appropriate for the comparative study of complex milk lipids *in vitro*. The treatment of ML_con _and ML_alp _for the production of an ethanolic FFA solution (100 mM) led to a significant loss of SCFA (short-chain fatty acid) and a decrease in MCFA (middle-chain fatty acid) levels that resulted in a redistribution and percentage increase of the majority of FA (Table 1B). However, independent of the treatment procedure, a fraction of the FA, namely stearic acid (C18:0), arachidic acid (C20:0), gondoic acid (C20:1 *c*11), and dihomo-γ-linolenic acid (C20:3 n-6) remained generally unaffected.

**Table 1:** Fatty acid distribution of milk lipids

	A								B							
	
	ML_con_				ML_alp_				ML_con_				ML_alp_			
**FAME [%]**	**Mean**		**SD**		**Mean**		**SD**		**Mean**		**SD**		**Mean**		**SD**	

C14:0	12.32	±	0.51	^**b**^	9.67	±	0.25	^**d**^	13.09	±	0.18	^**a**^	10.21	±	0.18	^**c**^
C14:1 *c*9	1.04	±	0.05	^**b**^	0.89	±	0.03	^**d**^	1.15	±	0.02	^**a**^	0.98	±	0.01	^**c**^
C15:0	1.24	±	0.05	^**d**^	1.35	±	0.02	^**c**^	1.47	±	0.04	^**b**^	1.58	±	0.04	^**a**^
C16:0	38.36	±	1.29	^**b**^	26.49	±	0.89	^**d**^	43.50	±	1.35	^**a**^	29.76	±	0.53	^**c**^
C16:1 *c*9	1.70	±	0.05	^**b**^	1.37	±	0.04	^**d**^	1.97	±	0.01	^**a**^	1.62	±	0.04	^**c**^
C17:0	0.56	±	0.02	^**c**^	0.68	±	0.01	^**b**^	0.68	±	0.02	^**b**^	0.79	±	0.01	^**a**^
C18:0	9.53	±	1.07		10.19	±	0.85		9.92	±	0.22		10.54	±	0.26	
C18:1 *c*9	14.06	±	0.65	^**d**^	19.79	±	0.68	^**b**^	15.56	±	0.19	^**c**^	21.71	±	0.27	^**a**^
C18:1 *c*11	0.46	±	0.02	^**d**^	0.60	±	0.02	^**b**^	0.50	±	0.02	^**c**^	0.66	±	0.02	^**a**^
C18:1 *c*12-15	0.40	±	0.02	^**b**^	0.55	±	0.03	^**a**^	0.41	±	0.03	^**b**^	0.56	±	0.05	^**a**^
C18:1 *t*4-8	0.09	±	0.01	^**b**^	0.25	±	0.02	^**a**^	0.09	±	0.02	^**b**^	0.25	±	0.01	^**b**^
C18:1 *t*9	0.16	±	0.01	^**b**^	0.29	±	0.01	^**a**^	0.15	±	0.03	^**b**^	0.30	±	0.01	^**a**^
C18:1 *t*10	0.21	±	0.02	^**c**^	0.28	±	0.02	^**b**^	0.22	±	0.02	^**c**^	0.32	±	0.01	^**a**^
C18:1 *t*11 (VA)	0.47	±	0.02	^**c**^	4.54	±	0.13	^**b**^	0.51	±	0.01	^**c**^	4.96	±	0.05	^**a**^
C18:1 *t*12-16	0.81	±	0.07	^**c**^	1.25	±	0.10	^**b**^	0.91	±	0.03	^**c**^	1.44	±	0.04	^**a**^
C18:2 n-6	1.06	±	0.09	^**b**^	1.30	±	0.06	^**ab**^	1.48	±	0.43	^**ab**^	1.73	±	0.38	^**a**^
C18:2 n-6 (*t*,*t*)	0.30	±	0.02	^**c**^	0.52	±	0.04	^**b**^	0.35	±	0.03	^**c**^	0.71	±	0.06	^**a**^
C18:2 *c*9,*t*11	0.31	±	0.03	^**c**^	2.77	±	0.13	^**b**^	0.32	±	0.04	^**c**^	3.04	±	0.08	^**a**^
C18:2 *t*11,*c*13	0.00	±	0.00	^**c**^	0.25	±	0.01	^**b**^	0.00	±	0.00	^**c**^	0.29	±	0.02	^**a**^
C18:2 *t*9,*t*11	0.03	±	0.01	^**c**^	0.12	±	0.00	^**b**^	0.01	±	0.01	^**d**^	0.16	±	0.02	^**a**^
C18:2 *t*11,*t*13	0.00	±	0.00	^**c**^	0.05	±	0.00	^**a**^	0.00	±	0.00	^**c**^	0.04	±	0.01	^**b**^
C18:3 n-3	0.34	±	0.03	^**d**^	1.27	±	0.06	^**b**^	0.43	±	0.03	^**c**^	1.46	±	0.02	^**a**^
C20:0	0.21	±	0.11		0.17	±	0.02		0.20	±	0.04		0.19	±	0.03	
C20:1 *c*9	0.02	±	0.00		0.03	±	0.01		0.04	±	0.02		0.03	±	0.02	
C20:3 n-6	0.09	±	0.01		0.07	±	0.01		0.09	±	0.03		0.07	±	0.02	
C20:4 n-6	0.10	±	0.01	^**a**^	0.07	±	0.00	^**b**^	0.10	±	0.01	^**a**^	0.08	±	0.02	^**b**^
C20:5 n-3	0.04	±	0.01	^**b**^	0.11	±	0.01	^**a**^	0.03	±	0.02	^**b**^	0.10	±	0.02	^**a**^
C22:5 n-3	0.09	±	0.01	^**b**^	0.12	±	0.00	^**ab**^	0.08	±	0.07	^**b**^	0.16	±	0.05	^**a**^
∑ SCFA(C4 > C8)	6.58	±	0.50	^**a**^	6.56	±	0.40	^**a**^	0.00	±	0.00	^**b**^	0.00	±	0.00	^**b**^
∑ MCFA(C10 > C14)	20.77	±	0.98	^**a**^	16.15	±	0.43	^**c**^	18.50	±	0.95	^**b**^	14.19	±	0.45	^**d**^
∑ SFA	77.73	±	1.00	^**a**^	62.95	±	1.17	^**c**^	75.25	±	0.74	^**b**^	58.98	±	0.35	^**d**^
∑ MUFA	19.89	±	0.81	^**d**^	30.29	±	0.93	^**b**^	21.67	±	0.21	^**c**^	33.02	±	0.33	^**a**^
∑ PUFA incl. CLA	2.38	±	0.19	^**d**^	6.76	±	0.25	^**b**^	3.07	±	0.58	^**c**^	8.01	±	0.38	^**a**^
∑ PUFA n-3	0.48	±	0.05	^**c**^	1.58	±	0.06	^**b**^	0.56	±	0.11	^**c**^	1.83	±	0.05	^**a**^
∑ PUFA n-6	1.26	±	0.10	^**b**^	1.46	±	0.06	^**ab**^	1.83	±	0.48	^**a**^	1.92	±	0.38	^**a**^
PUFA/SFA	0.03	±	0.00	^**d**^	0.11	±	0.01	^**b**^	0.04	±	0.01	^**c**^	0.14	±	0.01	^**a**^
n-6/n-3	2.60	±	0.12	^**b**^	0.93	±	0.01	^**c**^	3.26	±	0.23	^**a**^	1.05	±	0.19	^**c**^
∑ SFA+*t*FA	79.78	±	0.86	^**a**^	70.08	±	0.90	^**c**^	77.49	±	0.75	^**b**^	66.96	±	0.30	^**d**^
∑ C18:1 (*t*)	1.74	±	0.13	^**c**^	6.61	±	0.26	^**b**^	1.88	±	0.09	^**c**^	7.28	±	0.07	^**a**^
C18:1 *t*9/*t*11	0.35	±	0.01	^**a**^	0.06	±	0.00	^**c**^	0.30	±	0.05	^**b**^	0.06	±	0.00	^**c**^
∑ CLA	0.34	±	0.03	^**c**^	3.19	±	0.13	^**b**^	0.33	±	0.04	^**c**^	3.55	±	0.09	^**a**^
VA + C18:2 *c*9,*t*11	0.78	±	0.04	^**c**^	7.31	±	0.26	^**b**^	0.83	±	0.04	^**c**^	8.00	±	0.13	^**a**^
∑ BCFA	1.75	±	0.05	^**d**^	2.39	±	0.08	^**b**^	2.11	±	0.06	^**c**^	2.80	±	0.03	^**a**^

FA distribution of milk lipids from TAG (ML conventional and ML Alpine) before preparation (**A**) to FFA-mixtures suitable for cellular experiments, FA distribution of milk lipids as FFA-mixtures used for incubation of cells after saponification, extraction and solution (**B**) of milk lipids (FFA) in ethanol. Means (n = 6) of **FAME [%] **significantly differ without the same letter ^(**a**, **b**, **c**, **d**)^, two-way ANOVA Tukey *post hoc *test *P *< 0.05.

The measurement with Ag^+^-HPLC ought to provide information relating to CLA isomer distribution and may help to elucidate the consequences of the treatment (Table 2). The distribution of CLA isomers differed significantly. As determined by GC analysis, *c*9,*t*11-CLA was the major isomer. The portion of this isomer with regards to CLA distribution was higher for ML_alp _compared to ML_con _(Tables 1 and 2). The second most abundant CLA isomers of ML_con _and ML_alp _were *t*7,*c*9-CLA (9%) and *t*11,*c*13-CLA (8%), respectively. The individual ratios of *t*11,*c*13-CLA/*t*7,*c*9-CLA significantly differed by a factor of 17 and indicated an Alpine origin of the lipids as well as grazing on mountain pasture, as shown earlier [10]. Notably, the fraction of *all-cis *and *all*-*trans*-CLA of ML_con _was significantly higher compared to ML_alp_. Significant changes of the CLA profile during sample preparation of milk lipids to FFA-ML_con/alp _were not observed (Table 2).

**Table 2:** Fatty acid distribution of CLA isomers in different milk lipids

	A								B							
	
	ML_con_				ML_alp_				ML_con_				ML_alp_			
**CLA FAME [%]**	**Mean**		**SD**		**Mean**		**SD**		**Mean**		**SD**		**Mean**		**SD**	

*t*12,*t*14	2.36	±	0.08	^**a**^	0.96	±	0.01	^**b**^	2.34	±	0.16	^**a**^	1.00	±	0.02	^**b**^
*t*11,*t*13	4.19	±	0.09	^**a**^	1.86	±	0.05	^**b**^	4.05	±	0.16	^**a**^	1.92	±	0.06	^**b**^
*t*10,*t*12	0.95	±	0.07	^**a**^	0.17	±	0.04	^**b**^	0.97	±	0.09	^**a**^	0.33	±	0.09	^**b**^
*t*9,*t*11	2.68	±	0.24	^**b**^	0.54	±	0.03	^**c**^	3.27	±	0.16	^**a**^	0.85	±	0.20	^**c**^
*t*8,*t*10	1.41	±	0.08	^**a**^	0.19	±	0.03	^**b**^	1.53	±	0.21	^**a**^	0.22	±	0.09	^**b**^
*t*7,*t*9	0.42	±	0.03	^**a**^	0.07	±	0.03	^**b**^	0.41	±	0.10	^**a**^	0.06	±	0.03	^**b**^
∑ *trans*,*trans*	12.18	±	0.24	^**a**^	3.84	±	0.07	^**b**^	12.69	±	0.74	^**a**^	4.42	±	0.44	^**b**^
*t*11,*c*13	1.51	±	0.15	^**b**^	7.88	±	0.02	^**a**^	1.29	±	0.08	^**b**^	7.83	±	0.09	^**a**^
*c*11,*t*13	0.39	±	0.14		0.17	±	0.03		0.25	±	0.12		0.24	±	0.07	
*t*10,*c*12	0.51	±	0.03	^**ab**^	0.07	±	0.04	^**b**^	0.74	±	0.26	^**a**^	0.32	±	0.38	^**ab**^
*c*9,*t*11	74.46	±	0.59	^**b**^	84.57	±	0.19	^**a**^	74.64	±	0.63	^**b**^	83.77	±	0.86	^**a**^
*t*8,*c*10	0.91	±	0.25		0.60	±	0.13		1.21	±	0.28		0.61	±	0.27	
*t*7,*c*9	8.97	±	0.28	^**a**^	2.64	±	0.23	^**b**^	8.39	±	0.09	^**a**^	2.56	±	0.34	^**b**^
∑ *cis*,*trans*/*trans*,*cis*	86.90	±	0.25	^**b**^	95.99	±	0.02	^**a**^	86.62	±	0.75	^**b**^	95.39	±	0.42	^**a**^
*c*11,*c*13	0.07	±	0.04		0.10	±	0.06		0.10	±	0.06		0.09	±	0.03	
*c*10,*c*12	0.10	±	0.08		-				0.05	±	0.01		-			
*c*9,*c*11	0.67	±	0.20	^**a**^	0.07	±	0.01	^**c**^	0.54	±	0.30	^**ab**^	0.10	±	0.01	^**ab**^
*c*8,*c*10	-				-				-				-			
∑ *cis*/*cis*	0.84	±	0.08	^**a**^	0.17	±	0.06	^**b**^	0.69	±	0.33	^**a**^	0.18	±	0.02	^**b**^
*t*11,*c*13/*t*7,*c*9	0.17	±	0.02	^**b**^	3.00	±	0.26	^**a**^	0.15	±	0.01	^**b**^	3.10	±	0.48	^**a**^

FA distribution of milk lipids from TAG (ML conventional and ML Alpine) before preparation (**A**) to FFA-mixtures suitable for cellular experiments, FA distribution of milk lipids as FFA-mixtures used for incubation of cells after saponification, extraction and solution (**B**) of milk lipids (FFA) in ethanol. Means (n = 6) of **FAME [%] **significantly differ without the same letter ^(**a**, **b**, **c**, **d**)^, two-way ANOVA Tukey *post hoc *test *P *< 0.05.

### Fatty acid analysis of HT-29 cells

**Figure 1:**
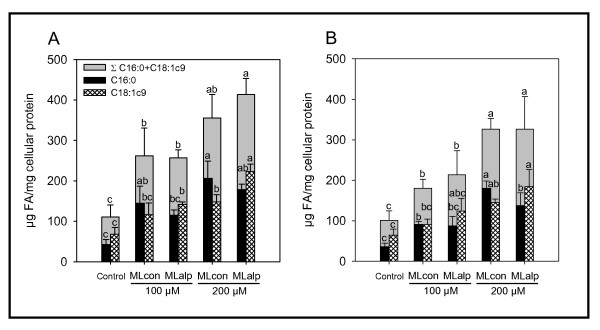
**Quantitative amounts of fatty acids in cells after treatment with milk lipids**. Total amount of two major FA of HT-29 cells after incubation with milk lipids (ML conventional and ML Alpine) given as FFA-mixtures in ethanol after 8 h (**A**) and 24 h (**B**) of incubation. Means (n = 6) of μg FA/mg cellular protein differ without the same letter ^(a, b, c)^, two-way ANOVA Tukey *post hoc *test *P *< 0.05.

Incubation of HT-29 cells with complex lipids in the form of FFA derivatives prerequisites an analysis of the degree of FA incorporation allowing evaluation of the influence of different FA profiles on cellular lipid metabolism. FFA Concentrations of 100 μM and 200 μM were chosen because preliminary experiments showed no significant influence of these values on the viability of HT-29 cells. Results revealed that treatment with ML_con/alp _led to a significant alteration of total cellular lipid content including FA distribution at 8 h and 24 h of incubation. Absolute quantitative determination showed a dose-dependent increase of the major FA C16:0 and C18:1 *c*9 (μg FA/mg cellular protein) at 8 h and 24 h of incubation (Figure 1). However, after 24 h of incubation the amount of total cellular lipids as measured for the two FA (μg FA/mg cellular protein) decreased in a time-dependent manner with the exception of C16:0 and C18:1 *c*9 after incubation with 200 μM of ML_con,_. Cellular lipid content of control did not change between 8 h and 24 h. On subtracting the lipid content of untreated cells (control), the treatment with ML_alp _and ML_con _led to a dose-dependent increase for both FA by 2 fold (ML_alp_; 8 h and 24 h of incubation), by 1.5 fold (ML_con_; 8 h) and by 3 fold (ML_con_; 24 h), respectively. Consequently, the main characteristics of the different milk lipids were reflected by these two important major FA. Thus, ML_con _(200 μM at 24 h) led to the significantly highest concentration of one SFA (C16:0), whereas, in contrast, ML_alp _caused the significantly highest concentration of one MUFA (C18:1 *c*9) (200 μM at 8 h). The sum of both FA reflected a dose-dependent increase (Figure 1).

Relative quantitative analysis supported this observation and showed a significant alteration of cell lipid composition (Tables 3 and 4). The percentage of SFA was considerably increased after incubation with the milk lipids compared to the control. Typically, ML_con _led to higher increase of SFA compared to ML_alp_. Otherwise, ML_alp _led to higher portions of MUFA compared to ML_con_, but not to the control. Additionally, PUFA, including CLA were significantly lower after 8 h of incubation with ML_con _compared to ML_alp _and the control, and further decreased after 24 h with ML_con _and control compared to ML_alp_. Notably, PUFA (n-3 and n-6) were lower after incubation with milk lipids compared to the control. However, ML_con _significantly increased whereas ML_alp _significantly decreased the ratio of n-6/n-3 PUFA. In addition, ML_alp _showed the highest dose-dependent portions of CLA, *trans*-C18:1 and BCFA in cellular lipids at 8 h and 24 h of incubation (Tables 3 and 4). Particularly, a doubling of the concentration did not lead to further changes of FA composition for either incubation procedures. C18:1 *t*11 converted to *c*9,*t*11-CLA dose- and time-dependently through Δ9-desaturation following incubation with ML_alp_. Generally, ML_alp _significantly increased total CLA compared to ML_con _by a factor of 8 (100 μM) and 9 (200 μM). The formation of conjugated metabolites was found only after incubation with ML_alp_. Despite the low percentage of total CLA lipid content, C16:2 *c*7,*t*9 increased dose- and time-dependently (Tables 3 and 4).

**Table 3:** Fatty acid distribution of cellular lipids treated with different milk lipids at 8 h

	Control				ML_con_								ML_alp_							
	
	0.25% EtOH				100 μM				200 μM				100 μM				200 μM			
**FAME [%]**	**Mean**		**SD**		**Mean**		**SD**		**Mean**		**SD**		**Mean**		**SD**		**Mean**		**SD**	

C14:0	1.58	±	0.39	^d^	6.38	±	0.87	^bc^	7.96	±	0.94	^a^	5.38	±	0.57	^c^	6.81	±	0.51	^ab^
C14:1 *c*9	0.10	±	0.04	^d^	0.24	±	0.05	^bc^	0.33	±	0.05	^a^	0.22	±	0.05	^c^	0.30	±	0.03	^ab^
C15:0	0.25	±	0.03	^d^	1.02	±	0.09	^bc^	1.18	±	0.08	^a^	1.08	±	0.09	^c^	1.28	±	0.07	^ab^
C16:0	20.92	±	0.95	^d^	34.53	±	1.89	^b^	36.96	±	1.35	^a^	26.52	±	1.14	^c^	26.77	±	0.99	^c^
C16:1 *c*9	12.75	±	0.74	^a^	8.11	±	0.80	^b^	6.64	±	0.52	^c^	7.11	±	0.51	^bc^	5.58	±	0.36	^d^
C16:2 *t*7,*c*9	-				-				-				0.03	±	0.01	^b^	0.05	±	0.01	^a^
C17:0	0.44	±	0.03	^c^	0.64	±	0.04	^b^	0.63	±	0.02	^b^	0.69	±	0.04	^a^	0.67	±	0.02	^ab^
C18:0	13.02	±	0.47	^a^	10.23	±	0.29	^b^	9.34	±	0.63	^c^	10.13	±	0.37	^b^	8.66	±	0.27	^c^
C18:1 *c*9	34.32	±	0.82	^a^	25.93	±	1.71	^c^	25.02	±	1.66	^c^	29.23	±	1.32	^b^	29.05	±	1.34	^b^
C18:1 *c*11	3.25	±	0.18	^a^	1.85	±	0.24	^b^	1.47	±	0.08	^c^	1.90	±	0.12	^b^	1.56	±	0.07	^c^
C18:1 *c*12-15	-				0.31	±	0.10	^b^	0.39	±	0.09	^ab^	0.42	±	0.13	^ab^	0.48	±	0.11	^a^
C18:1 *t*4-8	-				0.03	±	0.01	^bc^	0.03	±	0.01	^bc^	0.08	±	0.06	^ab^	0.11	±	0.05	^a^
C18:1 *t*9	-				0.10	±	0.03	^b^	0.10	±	0.04	^b^	0.16	±	0.04	^ab^	0.19	±	0.07	^a^
C18:1 *t*10	-				0.14	±	0.04	^b^	0.18	±	0.03	^b^	0.19	±	0.04	^b^	0.25	±	0.05	^a^
C18:1 *t*11 (VA)	-				0.27	±	0.03	^c^	0.34	±	0.03	^c^	2.50	±	0.20	^b^	3.32	±	0.16	^a^
C18:1 *t*12-16	-				0.42	±	0.14	^c^	0.54	±	0.18	^bc^	0.75	±	0.19	^ab^	0.92	±	0.20	^a^
C18:2 n-6	2.65	±	0.13	^a^	2.08	±	0.05	^cd^	2.04	±	0.17	^d^	2.28	±	0.07	^b^	2.25	±	0.11	^bc^
C18:2 n-6 (*t*,*t*)	-				0.21	±	0.06	^d^	0.29	±	0.03	^c^	0.43	±	0.04	^b^	0.54	±	0.03	^a^
C18:2 *c*9,*t*11	0.09	±	0.01	^e^	0.32	±	0.01	^d^	0.37	±	0.03	^c^	2.47	±	0.07	^b^	3.18	±	0.14	^a^
C18:2 *t*11,*c*13	-				-				-				0.18	±	0.02	^b^	0.26	±	0.02	^a^
C18:2 *t*9,*t*11	-				0.03	±	0.01	^cd^	0.04	±	0.01	^c^	0.18	±	0.02	^b^	0.24	±	0.03	^a^
C18:3 n-3	0.22	±	0.03	^d^	0.33	±	0.02	^c^	0.42	±	0.05	^c^	0.96	±	0.02	^b^	1.31	±	0.09	^a^
C20:0	0.38	±	0.03	^a^	0.30	±	0.02	^b^	0.24	±	0.03	^c^	0.28	±	0.02	^b^	0.20	±	0.02	^c^
C20:1 *c*9	0.33	±	0.07	^a^	0.17	±	0.04	^bc^	0.15	±	0.02	^d^	0.23	±	0.04	^b^	0.18	±	0.02	^bc^
C20:3 n-6	1.19	±	0.14	^a^	0.63	±	0.05	^b^	0.49	±	0.04	^cd^	0.58	±	0.06	^bc^	0.42	±	0.05	^d^
C20:4 n-6	2.38	±	0.25	^a^	1.20	±	0.09	^b^	0.86	±	0.05	^c^	1.13	±	0.13	^b^	0.77	±	0.09	^c^
C20:5 n-3	0.70	±	0.05	^a^	0.35	±	0.04	^bc^	0.27	±	0.02	^d^	0.39	±	0.03	^b^	0.31	±	0.03	^cd^
C22:5 n-3	1.34	±	0.15	^a^	0.70	±	0.11	^b^	0.53	±	0.06	^c^	0.69	±	0.08	^bc^	0.54	±	0.07	^bc^
C22:6 n-3	1.76	±	0.16	^a^	0.82	±	0.11	^b^	0.58	±	0.05	^c^	0.78	±	0.10	^b^	0.53	±	0.07	^c^
∑ SFA	38.65	±	0.88	^a^	55.47	±	2.59	^b^	58.67	±	1.83	^a^	46.78	±	1.59	^c^	47.29	±	1.66	^c^
∑ MUFA	50.84	±	0.34	^b^	37.69	±	2.28	^c^	35.30	±	1.49	^c^	42.91	±	1.34	^b^	42.07	±	1.22	^b^
∑ PUFA incl. CLA	10.51	±	0.86	^a^	6.84	±	0.47	^b^	6.03	±	0.44	^b^	10.31	±	0.44	^a^	10.64	±	0.56	^a^
∑ PUFA n-3	4.14	±	0.36	^a^	2.31	±	0.28	^c^	1.88	±	0.16	^c^	2.95	±	0.21	^b^	2.82	±	0.22	^b^
∑ PUFA n-6	6.28	±	0.51	^a^	3.98	±	0.18	^bc^	3.46	±	0.25	^d^	4.07	±	0.25	^b^	3.54	±	0.23	^cd^
PUFA/SFA	0.27	±	0.03	^a^	0.12	±	0.01	^c^	0.10	±	0.01	^c^	0.22	±	0.02	^b^	0.23	±	0.02	^b^
n-6/n-3	1.52	±	0.06	^b^	1.74	±	0.17	^a^	1.85	±	0.07	^a^	1.38	±	0.03	^bc^	1.26	±	0.02	^c^
∑ SFA + *t*FA	38.65	±	0.88	^d^	56.71	±	2.73	^b^	60.23	±	2.06	^a^	50.97	±	2.04	^c^	52.73	±	1.91	^c^
∑ C18:1 (*t*)	-				0.96	±	0.18	^c^	1.18	±	0.26	^c^	3.68	±	0.46	^b^	4.79	±	0.38	^a^
C18:1 *t*9,*t*11	-				0.37	±	0.10	^a^	0.28	±	0.09	^a^	0.06	±	0.01	^b^	0.06	±	0.02	^b^
∑ CLA	0.09	±	0.01	^d^	0.35	±	0.02	^c^	0.41	±	0.04	^c^	2.83	±	0.07	^b^	3.68	±	0.15	^a^
VA + C18:2 *c*9,*t*11	0.09	±	0.01	^d^	0.59	±	0.04	^c^	0.71	±	0.03	^c^	4.97	±	0.25	^b^	6.50	±	0.30	^a^
∑ BCFA	0.44	±	0.12	^d^	1.59	±	0.15	^c^	1.83	±	0.13	^b^	1.97	±	0.16	^b^	2.38	±	0.14	^a^

FA distribution of HT-29 cells after incubation with milk lipids (ML conventional and ML Alpine) given as FFA-mixtures in ethanol. Means (n = 6) of **FAME [%] **significantly differ without the same letter ^(**a**, **b**, **c**, **d**)^, two-way ANOVA Tukey *post hoc *test *P *< 0.05.

**Table 4:** Fatty acid distribution of cellular lipids treated with different milk lipids at 24 h

	Control				ML_con_								ML_alp_							
	
	0.25% EtOH				100 μM				200 μM				100 μM				200 μM			
**FAME [%]**	**Mean**		**SD**		**Mean**		**SD**		**Mean**		**SD**		**Mean**		**SD**		**Mean**		**SD**	

C14:0	1.67	±	0.32	^**d**^	6.26	±	0.34	^**b**^	8.33	±	0.61	^**a**^	5.36	±	0.28	^**c**^	6.86	±	0.46	^**b**^
C14:1 *c*9	0.17	±	0.06	^**c**^	0.55	±	0.09	^**ab**^	0.67	±	0.15	^**a**^	0.46	±	0.06	^**b**^	0.54	±	0.07	^**ab**^
C15:0	0.21	±	0.01	^**c**^	0.81	±	0.06	^**b**^	1.04	±	0.04	^**a**^	0.85	±	0.04	^**b**^	1.12	±	0.05	^**a**^
C16:0	20.48	±	0.71	^**d**^	30.34	±	1.27	^**b**^	34.36	±	1.34	^**a**^	24.56	±	0.65	^**c**^	25.57	±	0.29	^**c**^
C16:1 *c*9	15.44	±	0.97	^**a**^	11.82	±	1.23	^**b**^	9.45	±	1.09	^**c**^	9.31	±	0.95	^**c**^	6.96	±	0.73	^**d**^
C16:2 *t*7,*c*9	-				-				-				0.07	±	0.01	^**b**^	0.08	±	0.00	^**a**^
C17:0	0.32	±	0.01	^**a**^	0.54	±	0.04	^**c**^	0.59	±	0.03	^**b**^	0.59	±	0.04	^**b**^	0.68	±	0.03	^**d**^
C18:0	10.97	±	0.89	^**a**^	9.39	±	0.32	^**b**^	8.77	±	0.34	^**b**^	9.03	±	0.21	^**b**^	8.71	±	0.25	^**b**^
C18:1 *c*9	36.95	±	0.87	^**a**^	28.67	±	0.64	^**d**^	26.09	±	0.27	^**e**^	31.63	±	0.99	^**b**^	30.27	±	0.34	^**c**^
C18:1 *c*11	3.04	±	0.20	^**a**^	1.77	±	0.15	^**b**^	1.33	±	0.09	^**a**^	1.84	±	0.05	^**b**^	1.39	±	0.11	^**a**^
C18:1 *c*12-15	-				0.29	±	0.15	^**a**^	0.38	±	0.07	^**a**^	0.41	±	0.13	^**a**^	0.43	±	0.10	^**a**^
C18:1 *t*4-8	-				0.02	±	0.01	^**bc**^	0.04	±	0.02	^**bc**^	0.07	±	0.04	^**ab**^	0.10	±	0.05	^**a**^
C18:1 *t*9	-				0.09	±	0.02	^**c**^	0.12	±	0.03	^**bc**^	0.15	±	0.04	^**ab**^	0.20	±	0.06	^**a**^
C18:1 *t*10	-				0.10	±	0.02	^**b**^	0.14	±	0.03	^**b**^	0.14	±	0.04	^**b**^	0.20	±	0.03	^**a**^
C18:1 *t*11 (VA)	-				0.18	±	0.04	^**c**^	0.28	±	0.05	^**c**^	1.86	±	0.25	^**b**^	2.73	±	0.27	^**a**^
C18:1 *t*12-16	-				0.35	±	0.09	^**c**^	0.58	±	0.08	^**b**^	0.56	±	0.13	^**b**^	0.81	±	0.18	^**a**^
C18:2 n-6	2.13	±	0.18	^**a**^	1.91	±	0.12	^**bc**^	1.73	±	0.07	^**c**^	2.13	±	0.09	^**a**^	2.00	±	0.04	^**ab**^
C18:2 n-6 (*t*,*t*)	-				0.13	±	0.05	^**d**^	0.22	±	0.03	^**c**^	0.28	±	0.05	^**b**^	0.42	±	0.03	^**a**^
C18:2 *c*9,*t*11	0.07	±	0.02	^**d**^	0.35	±	0.02	^**c**^	0.40	±	0.02	^**c**^	2.90	±	0.27	^**b**^	3.57	±	0.18	^**a**^
C18:2 *t*11,*c*13	-				-				-				0.15	±	0.02	^**b**^	0.21	±	0.02	^**a**^
C18:2 *t*9,*t*11	-				0.04	±	0.01	^**c**^	0.04	±	0.01	^**c**^	0.19	±	0.02	^**b**^	0.21	±	0.02	^**a**^
C18:3 n-3	0.20	±	0.03	^**d**^	0.35	±	0.03	^**c**^	0.39	±	0.03	^**c**^	1.03	±	0.09	^**b**^	1.26	±	0.06	^**a**^
C20:0	0.35	±	0.02	^**a**^	0.30	±	0.02	^**b**^	0.28	±	0.01	^**bc**^	0.30	±	0.01	^**b**^	0.27	±	0.01	^**c**^
C20:1 *c*9	0.41	±	0.05	^**a**^	0.19	±	0.04	^**cd**^	0.15	±	0.00	^**d**^	0.26	±	0.02	^**b**^	0.21	±	0.01	^**bc**^
C20:3 n-6	0.97	±	0.12	^**a**^	0.55	±	0.01	^**b**^	0.38	±	0.01	^**c**^	0.53	±	0.02	^**b**^	0.36	±	0.02	^**c**^
C20:4 n-6	1.82	±	0.19	^**a**^	1.00	±	0.02	^**b**^	0.66	±	0.03	^**c**^	0.95	±	0.04	^**b**^	0.63	±	0.04	^**c**^
C20:5 n-3	0.53	±	0.05	^**a**^	0.29	±	0.02	^**bc**^	0.21	±	0.01	^**d**^	0.33	±	0.02	^**b**^	0.25	±	0.01	^**cd**^
C22:5 n-3	1.04	±	0.15	^**a**^	0.59	±	0.03	^**b**^	0.42	±	0.01	^**c**^	0.62	±	0.05	^**b**^	0.43	±	0.03	^**c**^
C22:6 n-3	1.31	±	0.12	^**a**^	0.70	±	0.03	^**b**^	0.45	±	0.03	^**c**^	0.70	±	0.05	^**b**^	0.43	±	0.04	^**c**^
∑ SFA	35.66	±	1.27	^**e**^	49.81	±	1.52	^**b**^	55.66	±	1.15	^**a**^	43.14	±	1.00	^**d**^	45.98	±	0.53	^**c**^
∑ MUFA	56.10	±	1.15	^**a**^	44.15	±	1.60	^**c**^	39.35	±	1.17	^**d**^	46.84	±	0.86	^**b**^	43.99	±	0.63	^**c**^
∑ PUFA incl. CLA	8.24	±	0.78	^**b**^	6.04	±	0.20	^**c**^	4.99	±	0.06	^**d**^	10.05	±	0.57	^**a**^	10.03	±	0.17	^**a**^
∑ PUFA n-3	3.20	±	0.34	^**a**^	2.01	±	0.07	^**d**^	1.51	±	0.04	^**e**^	2.78	±	0.17	^**b**^	2.47	±	0.04	^**c**^
∑ PUFA n-6	4.97	±	0.47	^**a**^	3.51	±	0.13	^**b**^	2.82	±	0.06	^**c**^	3.67	±	0.13	^**b**^	3.07	±	0.04	^**c**^
PUFA/SFA	0.23	±	0.03	^**a**^	0.12	±	0.00	^**b**^	0.09	±	0.00	^**c**^	0.23	±	0.02	^**a**^	0.22	±	0.00	^**a**^
n-6/n-3	1.56	±	0.07	^**c**^	1.75	±	0.07	^**b**^	1.87	±	0.07	^**a**^	1.32	±	0.03	^**d**^	1.25	±	0.01	^**d**^
∑ SFA + *t*FA	35.66	±	1.27	^**d**^	50.75	±	1.62	^**b**^	57.13	±	1.33	^**a**^	46.28	±	1.17	^**c**^	50.56	±	1.04	^**b**^
∑ C18:1 (*t*)	-				0.74	±	0.16	^**c**^	1.16	±	0.18	^**c**^	2.78	±	0.45	^**b**^	4.05	±	0.55	^**a**^
C18:1 *t*9/*t*11	-				0.51	±	0.07	^**a**^	0.42	±	0.07	^**b**^	0.08	±	0.02	^**c**^	0.07	±	0.02	^**c**^
∑ CLA	0.07	±	0.02	^**d**^	0.39	±	0.02	^**c**^	0.44	±	0.02	^**c**^	3.26	±	0.33	^**b**^	3.99	±	0.17	^**a**^
VA + C18:2 *c*9,*t*11	0.07	±	0.02	^**d**^	0.52	±	0.05	^**cd**^	0.68	±	0.06	^**c**^	4.75	±	0.52	^**b**^	6.30	±	0.40	^**a**^
∑ BCFA	0.37	±	0.10	^**d**^	1.36	±	0.13	^**c**^	1.68	±	0.10	^**b**^	1.72	±	0.17	^**b**^	2.16	±	0.11	^**a**^

FA distribution of HT-29 cells after incubation with milk lipids (ML conventional and ML Alpine) given as FFA-mixtures in ethanol. Means (n = 6) of **FAME [%] **significantly differ without the same letter ^(**a**, **b**, **c**, **d**)^, two-way ANOVA Tukey *post hoc *test *P *< 0.05.

The CLA composition of cellular lipids after incubation with the respective milk lipids was investigated using Ag^+^-HPLC (Table 5). Results showed the main characteristic portions of CLA after 8 h of incubation to be similar as compared to the distribution of CLA in intact milk lipids (Tables 2 and 5). Thus, ML_con_-treated cells showed highest portions of *all-trans*-CLA and *all-cis*-CLA as well as decreased levels of *cis*/*trans, trans*/*cis*-CLA compared to the control and ML_alp_. The ratio of *t*11,*c*13-CLA/*t*7,*c*9-CLA significantly differed after treatment with milk lipids as mentioned above for the distribution of ML_con/alp _(Tables 2 and 5).

**Table 5:** CLA isomers in cellular lipids of HT-29 treated with different milk lipids at 8 h

	Control				ML_con_								ML_alp_							
	
	0.25% EtOH				100 μM				200 μM				100 μM				200 μM			
**CLA FAME [%]**	**Mean**		**SD**		**Mean**		**SD**		**Mean**		**SD**		**Mean**		**SD**		**Mean**		**SD**	

*t*12,*t*14	-				-				-				-				-			
*t*11,*t*13	-				2.63	±	0.71	^**a**^	2.76	±	0.26	^**a**^	1.26	±	0.14	^**b**^	1.31	±	0.06	^**b**^
*t*10,*t*12	-				0.69	±	0.23	^**a**^	0.82	±	0.09	^**a**^	0.24	±	0.09	^**b**^	0.26	±	0.09	^**b**^
*t*9,*t*11	3.15	±	1.28	^**a**^	3.08	±	0.49	^**a**^	3.35	±	0.51	^**a**^	1.18	±	0.35	^**b**^	1.14	±	0.34	^**b**^
*t*8,*t*10	-				0.28	±	0.15	^**ab**^	0.47	±	0.16	^**a**^	0.02	±	0.01	^**bc**^	0.11	±	0.02	^**bc**^
*t*7,*t*9	2.86	±	0.79	^**a**^	0.89	±	0.28	^**b**^	0.73	±	0.08	^**b**^	0.09	±	0.05	^**b**^	0.05	±	0.01	^**b**^
∑ *trans, trans*	6.02	±	2.06	^**bc**^	8.38	±	1.19	^**ab**^	9.68	±	0.30	^**a**^	3.48	±	0.53	^**c**^	3.57	±	0.50	^**c**^
*t*11,*c*13	4.88	±	3.15	^**ab**^	3.59	±	1.20	^**ab**^	1.81	±	0.30	^**b**^	6.54	±	0.47	^**a**^	6.83	±	0.34	^**a**^
*c*11,*t*13	3.00	±	1.89	^**a**^	1.80	±	0.93	^**ab**^	1.17	±	0.25	^**ab**^	0.31	±	0.06	^**b**^	0.36	±	0.10	^**b**^
*t*10,*c*12	3.82	±	1.39	^**a**^	3.48	±	1.11	^**b**^	1.61	±	0.75	^**ab**^	0.42	±	0.27	^**b**^	0.22	±	0.15	^**b**^
*c*9,*t*11	77.28	±	3.25	^**b**^	74.65	±	0.80	^**a**^	76.12	±	0.98	^**b**^	86.66	±	1.17	^**a**^	87.14	±	0.80	^**a**^
*t*8,*c*10	0.63	±	0.45	^**ab**^	0.78	±	0.58	^**ab**^	1.20	±	0.25	^**a**^	0.44	±	0.10	^**ab**^	0.18	±	0.10	^**b**^
*t*7,*c*9	4.38	±	1.55	^**bc**^	6.09	±	1.58	^**cd**^	7.18	±	0.55	^**a**^	1.82	±	0.20	^**cd**^	1.44	±	0.11	^**d**^
∑ *cis*,*trans*/*trans*,*cis*	93.98	±	2.06	^**a**^	90.39	±	1.23	^**b**^	89.09	±	0.38	^**b**^	96.19	±	0.61	^**a**^	96.17	±	0.59	^**a**^
*c*11,*c*13	-				-				-				0.08	±	0.02		0.03	±	0.00	
*c*10,*c*12	-				-				-				-				-			
*c*9,*c*11	-				1.23	±	0.10	^**a**^	1.18	±	0.09	^**a**^	0.26	±	0.08	^**b**^	0.23	±	0.09	^**b**^
*c*8,*c*10	-				-				-				-				-			
∑ *cis*/*cis*	-				1.23	±	0.10	^**a**^	1.18	±	0.09	^**a**^	0.34	±	0.09	^**b**^	0.26	±	0.09	^**b**^
*t*11,*c*13/*t*7,*c*9	1.33	±	1.25	^**b**^	0.64	±	0.34	^**b**^	0.25	±	0.06	^**b**^	3.63	±	0.46	^**a**^	4.77	±	0.55	^**a**^

CLA distribution of HT-29 cells after incubation with milk lipids (ML conventional and ML Alpine) given as FFA-mixtures in ethanol. Means (n = 6) of **FAME [%] **significantly differ without the same letter ^(**a**, **b**, **c**, **d**)^, two-way ANOVA Tukey *post hoc *test *P *< 0.05.

### Effects of milk lipids on viability and growth

**Figure 2:**
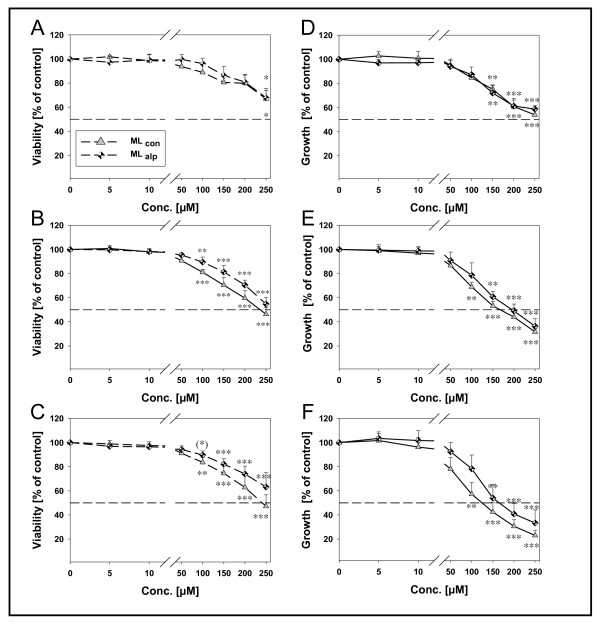
**Effects of milk lipids on viability and growth of HT-29 cells**. Cell viability was measured by means of the CTB assay (left; **A **24 h, **B **48 h, **C **72 h) and growth parameter of the cells was measured by means of DAPI assay (right; **D **24 h, **E **48 h, **F **72 h). Means (n = 6) are significantly different from control, one-way ANOVA Dunnett *post hoc *test (*) *P *< 0.1, * *P *< 0.05, ** *P *< 0.001, *** *P *< 0.0001.

The effects of lipids from two different milk sources (ML_alp_, ML_con_) on cell viability (CTB assay) and modulation of growth parameters (DAPI assay) were measured after incubation for 24 h, 48 h and 72 h, respectively (Figure 2). Viability and growth of HT-29 cells were reduced in a dose- and time-dependent manner. In particular, concentrations of 5 μM - 50 μM showed no effects compared to control. ML_con _and ML_alp _significantly reduced cell growth after treatment with 150 μM (24 h) to 75% and 72%, respectively. Viability was significantly decreased after 250 μM at 24 h of incubation. After 48 h, both lipids caused a decrease of cell viability and growth at 150 μM. After 48 h and 72 h of incubation, this was also observed for ML_con _at 100 μM (Figure 2). There were no significant differences between the milk lipids regarding the influence on viability and growth of HT-29 cells at any time point (Figure 2).

## Discussion

Naturally and synthetically derived CLA has been shown to act against artherogenesis, hypertension, diabetes, inflammation and cancer [26,27]. Whereas numerous studies indicate anticarcinogenic effects mostly due to single isomers, such as *c*9,*t*11-CLA or *t*10,*c*12-CLA [9,28] in different model systems, little is known about both the mode of action of isolated CLA isomers and their effectiveness when applied as a natural composition. Therefore, it is important to mimic the absorption of functional lipid ingredients in human gut with milk and dairy products. To test the potentially synergetic effects of FFA mixtures *in vitro*, HT-29 cells were treated with Alpine milk lipids richer in CLA, the precursor of CLA (C18:1 *t*11), and n-3 PUFA than conventional milk lipids.

First, we established an *in vitro *method that enabled us to test FFA mixtures of milk lipids. Alterations in milk lipid characteristics were monitored by this method. The SCFA fraction was excluded because levels of butyrate and other SCFA were not significantly different between the two types of milk, i.e., ML_alp_, ML_con _(Table 1). Furthermore, treatment with a mixture of PUFA and butyrate enhanced apoptosis of HT-29 cells [29]. Since the human gut is the main SCFA source of butyrate [30], we investigated potentially anticarcinogenic properties of milk fat *in vitro *considering only FA of chain lengths of over C8:0. Short saponification times (30 min) at a higher temperature (70°C) instead of the previously reported saponification conditions were more successful [31]. FFA were separated from total lipids and the prevention of oxidative artifacts, especially of the CLA isomers was proven by means of Ag^+^-HPLC analysis (Table 2). Although larger fractions of FA were enriched as a result of this method, the composition of the milk lipids was preserved and no effect on the distribution of important FA such as CLA isomers was detectable (Tables 1 and 2). Differences in the origin and type of feed were reflected by the respective milk lipid composition (Table 1). The results confirmed the typical FA distribution of ML_alp _and represented grazing food conditions in the Alps as described earlier [10,32]. Moreover, our results attested fatty acid distribution of ML_con _to intensive feeding conditions [11].

In addition to the fact that effects of a mixture of lipids of ruminant origin have been poorly studied *in vitro*, incorporation of milk lipid FA into cellular lipids has also been scantily investigated to date. However, the treatment of HT-29 cells involving 8 h and 24 h of incubation described herein verified not only the uptake and utilization of the FA mixtures, but also indicated a relationship of FA distribution and growth inhibition in HT-29 cells. Cell lipid content and FA composition were affected differently depending on treatment with ML_con _or ML_alp_. Whereas both milk lipids led to a comparable increase of total lipids, the ratio of the determined major FA (C16:0/C18:1 *c*9 μg FA/mg protein) differed between ML_con _and ML_alp _(Figure 1). By simply determining C16:0 and C18:1 *c*9 by means of GC-FID, the different origins of applied milk lipids and type of feeding could be distinguished. Acquisition of total major FA with total cellular protein yielded a decrease of FA after 24 h. Thus, a complete incorporation of milk lipids even after 8 h can be assumed (Figure 1).

Relative quantitative analysis of total cellular lipids following incubation with milk confirmed the high SFA proportion of total cellular lipids (Tables 3 and 4). Both milk lipids, ML_con _more than ML_alp_, led to an inversion of the SFA/MUFA ratio compared to control at 8 h. Notably, incubation with ML_alp _showed prolonged percentage of PUFA compared to ML_con _and control within cellular lipid distribution at 24 h. Generally, a doubling of the milk lipid concentration did not lead to a doubling of potentially favorable FA such as CLA and n-3 PUFA as shown by absolute quantitative analysis of C16:0 and C18:1 *c*9. This observation emphasizes the contrast to other investigations in which single purified isomers were used and showed a 2 fold increase of the administrated single FA in cellular lipids corresponding to the doubling of the FA concentration [33,34]. However, the absolute quantitative analysis in the present study showed a dose-dependent doubling of the major FA such as C16:0 and C18:1 *c*9. This important outcome strongly suggests that total CLA and other rumen-specific FA (C18:1 *t*11, BCFA) might be also doubled in a dose-dependent manner. Moreover, the results herein confirm that incubation of HT-29 cells with different milk lipids led to the incorporation of CLA into cellular lipids which remained consistent, although the majority of PUFA decreased time-dependently. A similar observation was made with C18:3 n-3 (Tables 3 and 4). Finally, the conversion of C18:1 *t*11 to *c*9,*t*11-CLA also contributed to an enhancement of total CLA in cellular lipids. Nevertheless, it remains questionable whether the daily consumption of Alpine milk and Alpine dairy products can raise the percentage of CLA into cellular lipids *in vivo *compared to *in vitro*. However, elevated amounts of total CLA and the CLA precursor C18:1 *t*11 in the tested ML_alp _can positively contribute to improve cellular levels of CLA *in vivo*. Thus, future human studies should further investigate if an equal intake of ML_alp _compared to ML_con _leads to 8 fold higher values of total CLA in cellular lipids as shown by the results of this study (Tables 3 and 4).

Anticancer activity of CLA was shown to be associated with modulation of cellular signal transduction [28,35,36]. Hence, it cannot be excluded that 8-9 fold lower amounts of CLA in ML_con _similarly influence cellular signaling pathways after incubation. Accordingly, these results can plausibly explain why a low concentration of between 0.1% and 1% CLA in the rodent diet was sufficient to produce anticarcinogenic effects [37,38].

The remarkable difference in the ratio of *t*11,*c*13-CLA/*t*7,*c*9-CLA was determined by means of Ag^+^-HPLC. This ratio can be used as a prospective marker for application of milk lipids with Alpine origin in a cellular model (Table 5). However, the influence of different CLA profiles and minor isomers such as *t*11,*c*13-CLA of Alpine milk on cellular lipid metabolism remains unclear.

Information on the interaction of conjugated fatty acids on FA metabolism is derived from the analysis of directly formed metabolites of CLA isomers (e.g. conjugated dienes; CD16:2, CD20:2, CD22:2) [39]. Analysis of these metabolites is important as it cannot be excluded that they also exhibit specific biological activity. For example, *c*9,*t*11-CLA can be metabolized to C16:2 *c*7,*t*9 by *β*-oxidation [39]. We found the metabolite C16:2 *c*7,*t*9 immediately following treatment with ML_alp _(Tables 3 and 4) at a percentage of below 0.1%. Moreover, the dose- and time-dependent *β*-oxidation of the major *c*9,*t*11-CLA isomer by HT-29 cells was evident (Tables 3 and 4). Although, it cannot be excluded that other metabolites could also be formed after incubation with ML_alp_, it is likely that concentrations of these metabolites would be extremely low.

Distribution of other cellular lipids was, moreover, affected by incubation with milk lipids. The dose- and time- dependent decrease of other PUFA such as C20:4 n6 (arachidonic acid) was shown in this study (Tables 3 and 4). This decrease must be interpreted as a dilution effect because milk lipids are naturally poor in LC-PUFA (> C20, long-chain). Stimulation experiments with milk lipids significantly decreased the percentage of n-6 as well as n-3 LC-PUFA (Tables 3 and 4) though absolute quantitative reduction remains questionable. Nevertheless, treatment with milk lipids [16] or CLA isomers [40] can partially inhibit conversion of LC-PUFA such as C20:4 n6 to eicosanoids, which are important FA derivatives that negatively affect tumorigenesis [41].

Viability and growth of the cells was reduced in a dose- and time-dependent manner on treatment with the two different milk lipids. However, there was no significant difference between the treatment with ML_con _and ML_alp _on these two parameters. Hence, it can be concluded that naturally occurring higher amounts of beneficial anticarcinogenic compounds like CLA, CLA precursor (C18:1 *t*11), and n-3 PUFA in Alpine milk fat did not increase anticarcinogenic activity of milk lipids in HT-29 cells at all. Generally, synergistic effects cannot be excluded when cells are treated with a mixture of lipids. Our results confirm data from an *in vitro *study of De La Torre *et al. *(2006) showing that a complete mixture of FA with low CLA content (< 1%) obtained from beef extract led to stronger growth inhibition of cancer cell lines compared to the fraction comprising of CLA-mixtures only. Indeed, FA from beef extract (100 μM) reduced cell numbers of different cell lines by 30-70% at 48 h of incubation [17] which was comparable to our study data. However, treatment of HT-29 cells with ML_alp _demonstrated an 8-9 fold increase of CLA that was not stronger compared to ML_con_. Notably, low concentrations (5 μM and 10 μM) showed no beneficial effects on proliferation of the cell line since C16:0 and C18:1 *c*9 are able to enhance cancer cell growth [42,43]. Physiological concentrations of total FFA *in vivo *are reported to range between 400 - 800 μM [44]. No data are available from the literature regarding the association between consumption of milk lipids and the resulting plasma concentration. In general, it is entirely conceivable that concentrations below 100 μM used herein could result from dietary intake of ruminant lipids. However, higher concentrations up to 250 μM emphasized the dose-effect relationship of the descriptive data from the *in vitro *experiments (Figure 2).

Other milk fat ingredients can also be responsible for the growth inhibitory effects found in this study. In particular, a health-beneficial potential was shown for BCFA (e.g. phytanic acid) in dairy lipids [49]. Treatment of colon cancer cells with ubiquitin and vitamin D, both constituents of milk fat, also led to inhibition of cell growth [50,51]. The repeated and continuous dietary intake of CLA *in vivo *showed elevated inhibitory effects on tumorigenesis [38]. It is, therefore, possible that the multiday incubation of ML_alp _at lower concentrations affect the growth of HT-29 cells differently compared to ML_con_. Further reasons for cell growth inhibition are likely to emerge, since CLA isomers are capable of inducing apoptosis and affecting cell cycle and DNA synthesis [36,52,53]. *All*-*trans*-CLA seem to exert increased growth inhibitory effects compared to CLA isomers with *cis *configuration [40,52-54]. Therefore, in further studies it will be of interest to determine whether FA mixtures rich in *all*-*trans*-CLA support this observation.

Several studies showed a dependency on anticarcinogenic effects *in vitro *[33] and *in vivo *[45-47] due to conversion of C18:1 *t*11 to CLA. This conversion of C18:1 *t*11 occurred immediately after treatment with ML_alp _in this study, but did not additionally contribute to growth inhibition compared to cells incubated with ML_con_, which were low in C18:1 *t*11 (Table 3). Furthermore, incubation with ML_con _markedly led to a significantly decreased PUFA/SFA ratio compared to control and ML_alp _treated cells (Table 4). The altered ratio was shown to be associated with increased apoptosis and inhibition of proliferation of PC-3 prostate cancer cells [48].

## Conclusions

This study showed significant changes of cell FA distribution and total amounts of cellular lipids after treatment with milk lipids of conventional or Alpine origin. Both milk lipid mixtures, in the form of FFA-solutions, effectively decreased viability and growth of HT-29 cells. However, concerning the inhibitory potential, no differences of these milk lipid mixtures were observed. Since the FA profile of the milk lipids determines the profile of the cell lipids, it might be promising to extend this feature to other cell lines. The intake of milk and dairy products is beneficial to health from the nutritional point of view, particularly with regards to colorectal cancer. However, mechanistic studies *in vitro *are required to distinguish between potential anticarcinogenic effects of single major isomers (*c*9,*t*11-CLA), single minor isomers (*t*11,*c*13-CLA), and complex lipids and to verify the efficacy in combination with other fatty acids and their corresponding matrix.

## Abbreviations

BCFA: branched-chain fatty acid; CLA: conjugated linoleic acid; FA: fatty acid; FAME: fatty acid methylesters; FFA: free fatty acids; HT-29: human colon adenocarcinoma cells; LC-PUFA: long-chain PUFA; MCFA: middle-chain fatty acid; ML_alp_: Alpine milk lipids; ML_con_: conventional milk lipids; MUFA: monounsaturated fatty acid; PBS: phosphate-buffered saline; PUFA: polyunsaturated fatty acid; SCFA: short-chain fatty acid; SFA: saturated fatty acid; TAG: triacylglycerides; VA: vaccenic acid

## Competing interests

The authors declare that they have no competing interests.

## Authors' contributions

CD conceived and carried out the study design, participated in the preparation of milk lipids, cell culture experiments, FA analysis by GC-FID, and performed Ag^+^-HPLC analysis, as well as conducting the determination of cell viability and growth, plus determining the cellular protein, analyzing the data, performing the statistical analysis and drafting the manuscript. AL participated in the study design and was involved in the preparation of milk lipids, cell culture experiments and absolute and relative quantitative FA analysis. SK participated in absolute quantitative FA analysis. KK participated in relative quantitative FA analysis with Ag^+^-HPLC. SD coordinated and performed a study with cows to provide conventional milk lipid samples. GJ helped to conceive the study, participated in its design and coordination and helped to draft the manuscript. All authors read and approved the final manuscript.
